# Modified Yoong Ultrasound‐Guided Rotator Interval Injection for Adhesive Capsulitis: A 2‐Year Follow‐Up

**DOI:** 10.1111/os.70405

**Published:** 2026-08-05

**Authors:** Azwan Aziz Mohamad, Norlelawati Mohamad, Muhammad Karbela Reza Ramlan, Nik Kamarul Arif, Badrul Akmal Hisham Md Yusoff

**Affiliations:** ^1^ Department of Orthopedic and Traumatology, Faculty of Medicine Universiti Kebangsaan Malaysia Cheras Wilayah Persekutuan Kuala Lumpur Malaysia

**Keywords:** adhesive capsulitis, articular, range of motion, shoulder pain, steroid

## Abstract

**Objective:**

Adhesive capsulitis (AC) is characterized by progressive fibrosis of the glenohumeral capsule, with the rotator interval (RI) as a key pathological site. Ultrasound‐guided hydrodilation of the RI (Yoong technique) is an established intervention, but traversal of a thickened coracohumeral ligament (CHL) can be technically challenging and painful. This study reports preliminary 2‐year outcomes of a modified technique targeting the subacromial–subdeltoid (SASD) bursa at the RI, combined with immediate postinjection manipulation.

**Methods:**

A retrospective case series was conducted on 13 patients with AC in the frozen phase treated between January 2023 and September 2023 at a single sports medicine clinic. The modified technique involved ultrasound‐guided SASD bursa injection (20 mL mixture: 4 mL 1% lidocaine, 15 mL 5% dextrose, 1 mL triamcinolone acetonide 40 mg) with mechanical fenestration of the CHL, followed by immediate shoulder manipulation. Outcomes included forward flexion, abduction, external rotation, Visual Analog Scale (VAS), and Quick DASH scores measured at baseline, 2, 12, 24, 52, and 104 weeks.

**Results:**

Mean age was 51.9 years (SD 7.52); 92.3% were female; 53.8% had diabetes. Significant improvements were observed across all outcomes (*p* < 0.001). Forward flexion improved from 76.6° (SD 6.3°) at baseline to 178.6° (SD 3.7°) at 104 weeks; abduction from 60.6° (SD 6.3°) to 178.8° (SD 2.7°); external rotation from 31.3° (SD 9.1°) to 78.0° (SD 3.6°). VAS decreased from 5.6 (SD 0.9) to 0 at 52 and 104 weeks. Quick DASH improved from 86.7 (SD 5.8) to 0.2 (SD 0.7) at 104 weeks. No complications were reported.

**Conclusion:**

This modified SASD‐targeted injection technique, combined with immediate manipulation, was associated with sustained pain relief and functional improvement over 2 years in a small cohort of AC patients.

## Graphical abstract

Adhesive capsulitis (frozen shoulder) in the frozen phase is characterized by thickening of the coracohumeral ligament (CHL) and fibrosis of the subacromial–subdeltoid (SASD) bursa. This study evaluated a modified ultrasound‐guided technique targeting the SASD bursa at the rotator interval, combined with mechanical CHL fenestration and immediate postinjection shoulder manipulation.

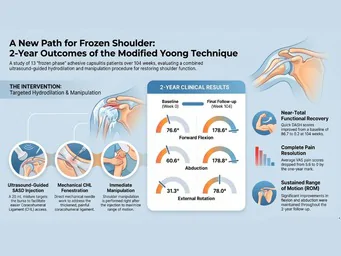

## 1 Introduction

Adhesive capsulitis (AC) is characterized by progressive fibrosis and inflammatory contraction of the glenohumeral joint capsule. Central to its pathology is the rotator interval (RI), a triangular space bordered by the subscapularis, supraspinatus, and coracoid process. This region houses the coracohumeral ligament (CHL) and superior glenohumeral ligament, which undergo significant morphological changes in AC. Magnetic resonance imaging (MRI) frequently demonstrates a thickened CHL (> 4 mm) and high signal intensity within the RI, reflecting active synovitis and capsular thickening [1]. For ultrasound, B‐mode ultrasound typically reveals three key features: thickening of the CHL (threshold ≥ 3.0 mm), thickening of the inferior capsule (cutoff 2.0–3.5 mm), and RI abnormalities such as hypoechoic soft tissue thickening or hypervascularity [2]. The ultrasound‐guided intra‐articular hydro‐dilation of the RI, popularized by Yoong et al. [3], has become a preferred intervention due to its direct targeting of the CHL pathology. However, the standard lateral‐to‐medial approach, which requires needle placement deep to the CHL, presents three distinct clinical challenges: (i) Tissue Resistance: In the frozen phase, the CHL is often profoundly thickened and fibrotic, making the injection physically difficult and acutely painful for the patient; (ii) Precision Risks: The narrow target zone increases the risk of accidental intra‐tendinous injection into the long head of the biceps or the supraspinatus; and (iii) Anatomical Crowding: A swollen supraspinatus often obscures the needle path, complicating precise tip placement.

Furthermore, fibrosis of the subacromial–subdeltoid (SASD) bursa is increasingly recognized as a key pathological feature of frozen shoulder, a condition described in the literature as adhesive bursopathy of the shoulder [4]. Sonographically, this manifests as nodular thickening of the synovial bursa, typically appearing as hypoechoic nodules. Consequently, ultrasound‐guided high‐volume distension of the SASD bursa has been proposed as a technique to release fibrotic bursal adhesions. The SASD bursa is densely innervated with free nerve endings, making it a primary generator of pain in AC. Interestingly, meta‐analyses have shown no significant difference in pain relief or range of motion (ROM) outcomes when comparing intra‐articular to subacromial corticosteroid injections, suggesting that addressing the extrinsic peritendinous environment is as vital as the intrinsic joint space [5]. The aim of this study is to report the 2‐year clinical outcomes of patients who underwent a modified Yoong technique for AC.

## 2 Methods

### 2.1 Study Design

This study was designed as a retrospective case series to evaluate the long‐term clinical efficacy of a modified Yoong ultrasound‐guided injection technique. Ethical approval for this study was granted by the Medical Research & Ethics Committee (NMRR‐21‐305‐58854). The study followed a cohort of patients diagnosed with the frozen phase of AC who were treated between January 1, 2023, and September 30, 2023.

### 2.2 Inclusion Criteria

Initial criteria highlighted for modified Yoong technique were a clinical diagnosis of primary (idiopathic) AC in the frozen phase, defined as follows: global restriction of passive and active shoulder ROM, with external rotation limitation > 50% compared to the contralateral side, duration of symptoms between 3 and 9 months, and normal plain radiograph of the shoulder (excluding glenohumeral arthritis, calcific tendinopathy, or fracture), with ultrasound confirmation of AC defined by at least two of the following three findings: (i) CHL thickening ≥ 3.0 mm, (ii) inferior capsular thickening ≥ 2.0 mm, (iii) RI abnormalities (hypoechoic soft tissue thickening and/or increased vascularity), and has failed conservative management for at least 6 weeks (including oral nonsteroidal anti‐inflammatory drugs and home exercise program) [2]. Patients currently receiving anticoagulant therapy were excluded from the procedure.

Data were retrospectively collected from secondary records at a Sports Medicine Clinic. Clinical outcomes were measured across the following parameters; pain Intensity assessed using the Visual Analog Scale (VAS), ROM; objective measurements of forward flexion and abduction were recorded, and Quick‐DASH score assessing the functional outcomes. Assessments were recorded at baseline, 2 weeks postinjection, and were recorded to a final 2‐year follow‐up during clinic assessments.

### 2.3 Technique

Key modification from previous technique is hydrodilation of SASD with fenestration of CHL, instead of glenohumeral injection. Prior to the procedure, the clinician performed an initial assessment of the patient's ROM and a standard ultrasound examination to evaluate the rotator cuff, RI, and subacromial bursa. A standardized sonographic protocol was used to scan the RI. With the patient supine and the shoulder slightly extended and externally rotated, the transducer was positioned to obtain a transverse oblique view of the RI, centering the long head of the biceps tendon (LHBT) between the supraspinatus (lateral) and subscapularis (medial) tendons (Video S1). In this view, the biceps reflection pulley, composed of the CHL draped superiorly over the LHBT and the superior glenohumeral ligament located medially, was accurately identified as the primary sonographic landmark for needle placement [6]. This precise anatomical localization ensured injection directly at the pain generator within the RI (Figure 1).

**FIGURE 1:**
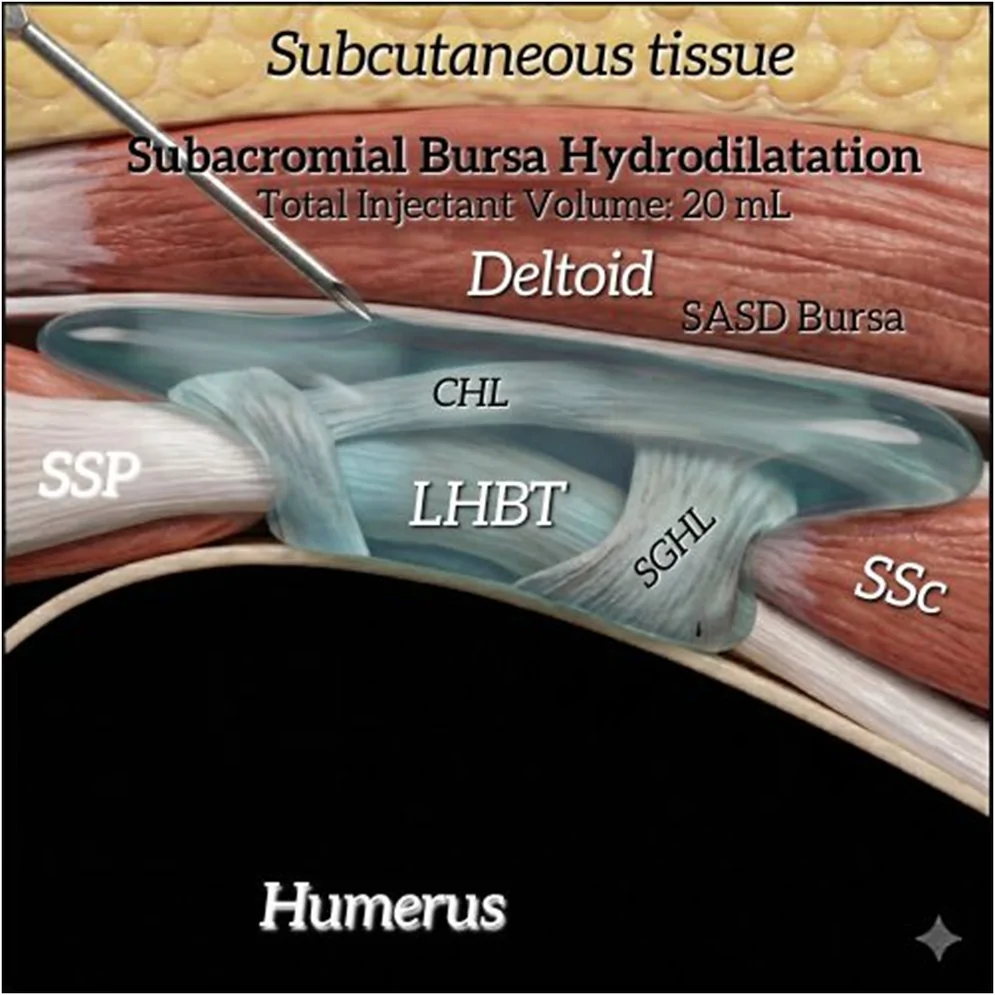
Illustration of Subacromial–subdeltoid hydrodistension at the rotator interval. CHL, coracohumeral ligament; LHBT, long head of biceps tendon; SASD, subacromial–subdeltoid bursae; SGHL, superior glenohumeral ligament; SSC, subscapularis; SSP, supraspinatus.

The interventional phase began with the subcutaneous infiltration of 1% lidocaine down to the SASD bursa using a 21‐gauge needle (Figure 2). The needle was subsequently advanced into the RI via an oblique, lateral‐to‐medial trajectory under real‐time ultrasound guidance. After the administration of local anesthetics, the interventionist performed mechanical fenestration of the thickened CHL to soften the tissue and facilitate injectate penetration. Following this, the needle tip was precisely positioned within the subacromial space, superior to the CHL. A 20‐mL bioactive mixture, consisting of 4 mL of 1% lidocaine, 15 mL of 5% dextrose, and 1 mL of triamcinolone acetonide (40 mg), was injected. Air bubbles were meticulously removed from the injectate to maintain optimal ultrasound visualization, ensuring that the fluid dispersed freely from the needle tip within the subacromial space.

**FIGURE 2:**
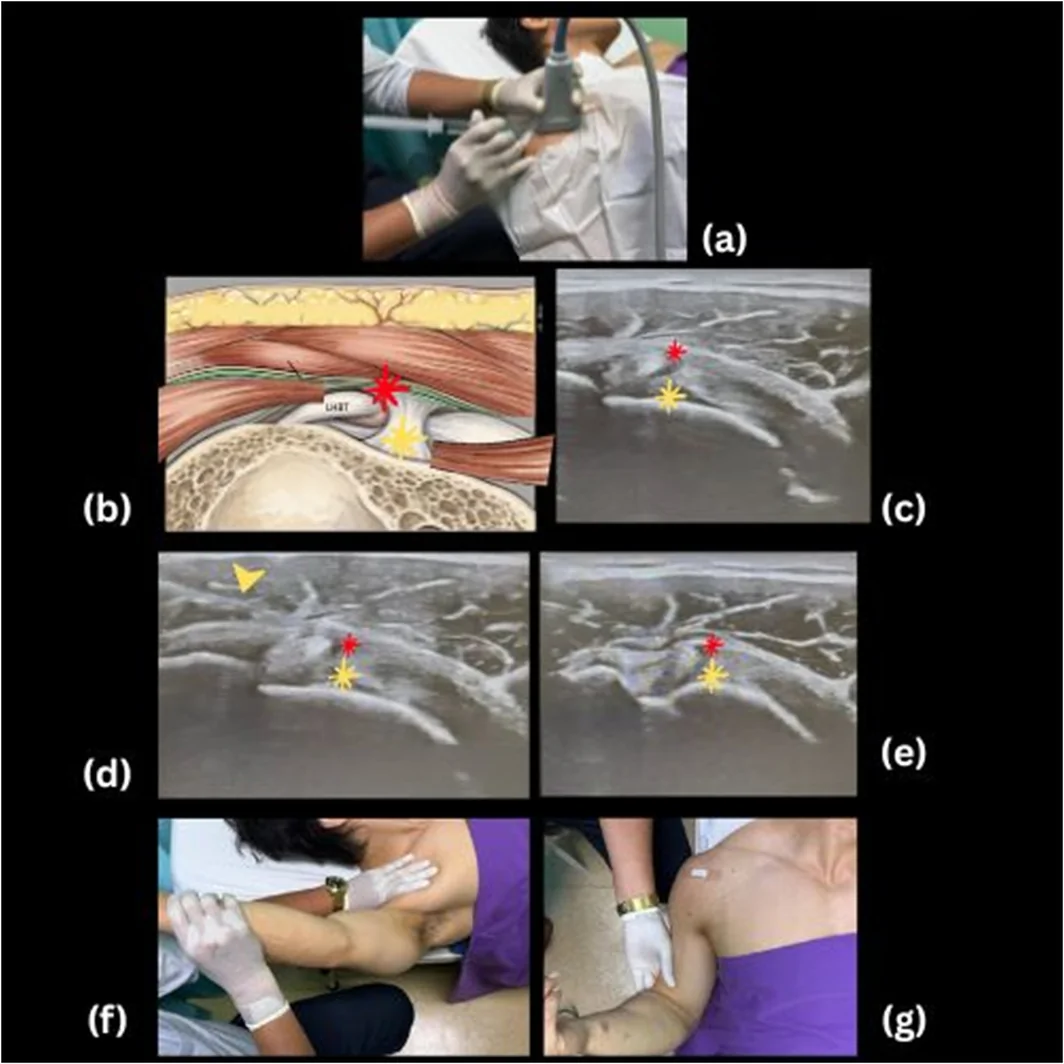
(a) With the patient lying on their back, the shoulder is extended and rotated outward while the elbow is bent to put the supraspinatus and subscapularis under tension. Place the probe obliquely, pointing toward the coracoid process. Using a lateral‐to‐medial approach, the injection is administered under sterile conditions. (b and c) Identify the corachohumeral ligament at the rotator interval in between the supraspinatus and subscapularis. (d) After infiltrating the subacromial–subdeltoid bursa with 3–5 mL of local anesthetic for pain relief and (e) the coracohumeral ligament is fenestrated using a 21‐gauge needle. The needle is then slightly withdrawn back into the bursa to deliver a 20 mL cocktail consisting of 1% lidocaine (4 mL), 5% dextrose (15 mL), and triamcinolone acetonide (1 mL/40 mg). Throughout the injection, the clinician should monitor for the “pumping effect”. (f) Immediately post injection, we performed shoulder manipulation in forward flexion, abduction, (g) and external rotation of shoulder. Yellow asterisk: superior glenohumeral ligament; Red asterisk: coracohumeral ligament; yellow arrow head: needle.

Immediate postprocedural care involved shoulder manipulation in forward flexion, abduction, and external rotation, followed by wall exercises. This was followed by a 20‐min application of an ice pack to manage postinjection inflammation. Finally, a referral to physiotherapy within 1 week was deemed a crucial component of the protocol to accelerate pain relief and the restoration of functional ROM.

### 2.4 Statistical Analysis

Data were analyzed using SPSS version 26.0 (IBM Corp., Armonk, NY, USA). Descriptive statistics were calculated for all baseline demographic and clinical characteristics. Continuous variables are presented as means with standard deviations (SD), and categorical variables as frequencies and percentages. To evaluate changes in outcome measures (forward flexion, abduction, external rotation, Visual Analog Scale [VAS], and Quick DASH scores) over the seven time points (baseline, 2, 12, 24, 52, and 104 weeks), a one‐way repeated‐measures analysis of variance (ANOVA) was conducted. Mauchly's test of sphericity was assessed for each dependent variable; when the assumption of sphericity was violated, Greenhouse–Geisser or Huynh–Feldt corrections were applied as appropriate to adjust the degrees of freedom. Statistical significance was set at a two‐tailed alpha level of *p* < 0.05. Post hoc pairwise comparisons using Bonferroni correction were planned to identify specific time points with significant differences, although the primary analysis focused on the overall time effect. Swimmer plot analysis was plotted for 13 patients against follow up. Resolution of AC was defined as the significant reduction of pain and the restoration of glenohumeral ROM to near‐normal or functional levels [7].

## 3 Results

### 3.1 Demographic Data

A retrospective preliminary analysis of secondary data was conducted on 13 patients with AC in the frozen phase, who were treated at the Sports Medicine Clinic between January 1, 2023, and September 30, 2023, and successfully underwent the technique. The mean age of the patients was 51.9 years ±7.52, with a predominantly female cohort (92.3%, *n* = 12). Underlying diabetes mellitus was present in 53.8% (*n* = 7) of the patients.

### 3.2 Outcome of Modified Yoong Technique

Significant improvements in shoulder ROM and Visual Analog Scale (VAS) scores were observed, as summarized in Table 1. At 2 weeks postinjection, all patients demonstrated significant gains in forward flexion and abduction. Subsequent follow‐ups revealed continued improvement in both ROM and pain levels through 104 weeks. No complications were reported. 92.3% had resolution of AC at 12 weeks (Figure 3).

**TABLE 1:** Clinical and functional assessment of the shoulder after modified Yoong procedures for 104 week.

	Baseline	2 weeks	12 weeks	24 weeks	52 weeks	104 weeks	Mauchly's	*F*	*p*
Forward flexion (°)	76.6 (6.3)	149.07 (10.7)	167.8 (5.9)	171.1 (2.5)	178.8 (2.9)	178.6 (3.7)	0.023	474.1	< 0.001
Abduction (°)	60.6 (6.3)	94.6 (9.8)	148.3 (6.2)	169.6 (5.2)	178.5 (2.8)	178.8 (2.7)	0.217	855.7	< 0.001
External rotation (°)	31.3 (9.1)	49.3 (9.7)	61.1 (8.3)	70.7 (4.4)	76.8 (3.9)	78 (3.6)	0.028	86.9	< 0.001
VAS	5.6 (0.9)	2 (0.9)	0.6 (0.7)	0.15 (0.3)	0 (0)	0 (0)	0.0	108.427	< 0.001
Quick DASH	86.7 (5.8)	47.3 (8.1)	22.7 (4.5)	5.5 (2.5)	0.3 (0.9)	0.2 (0.7)	0.005	750.485	< 0.001

*Note:* Repeated ANOVA was used to compare the outcomes measures.

Mean and standard deviation were reported during each follow up.

Mauchly's Test was used for sphericity; if value > 0.05, sphericity assumed, and if value < 0.05, Greenhouse–Geisser was used.

*p* < 0.05 indicates significant results.

**FIGURE 3:**
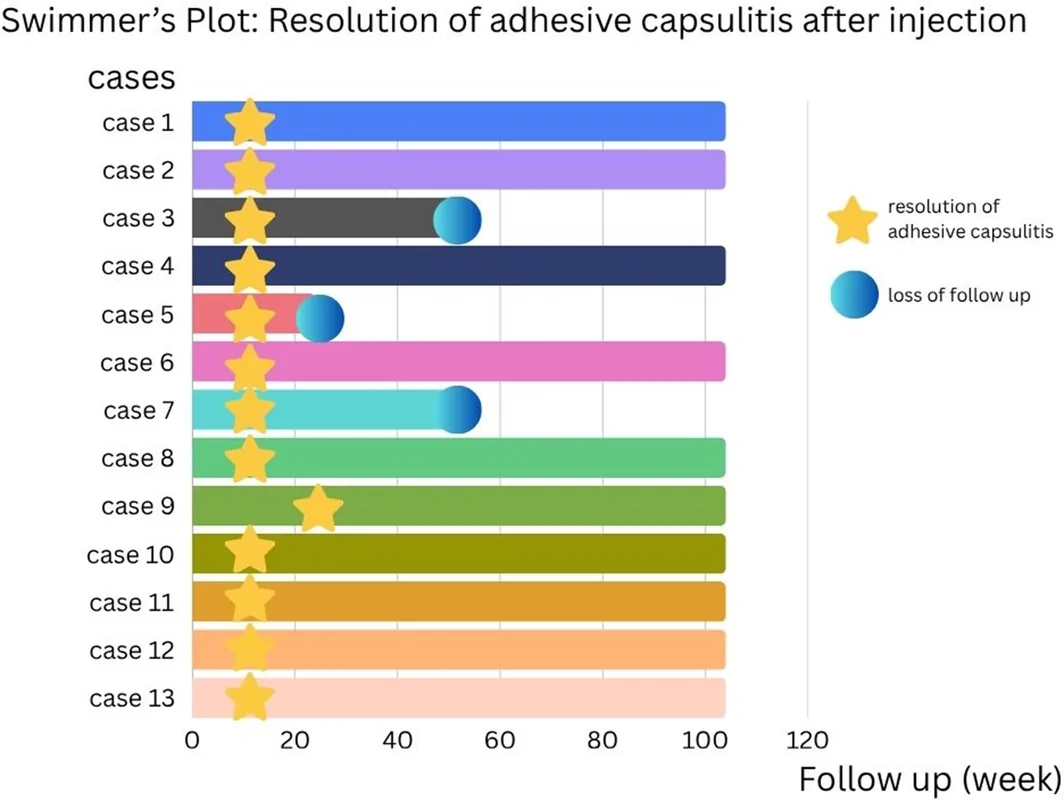
Swimmers plot of 13 patients follow up for 2 years. Twelve patients achieved resolution at 12 weeks post injection, while one patient achieved resolution at 24 weeks. Star indicates resolution of adhesive capsulitis. Circle indicates loss to follow up.

Repeated‐measures analysis (Table 1), with Mauchly's test of sphericity indicated where applicable, showed statistically significant changes over time for all outcome measures. Forward flexion improved from a baseline mean of 76.6° (SD = 6.3°) to 149.1° (SD = 10.7°) at 2 weeks, reaching 178.8° (SD = 2.9°) by 52 weeks. Abduction increased from 60.6° (SD = 6.3°) at baseline to 94.6° (SD = 9.8°) at 2 weeks, and further to 178.8° (SD = 2.7°) at 104 weeks (*F* = 855.7, *p* < 0.001). External rotation improved from 31.3° (SD = 9.1°) at baseline to 78.0° (SD = 3.6°) at 104 weeks (*F* = 86.9, *p* < 0.001). VAS scores decreased from 5.6 (SD = 0.9) at baseline to 0 at 52 and 104 weeks (*F* = 108.4, *p* < 0.001). Similarly, Quick DASH scores improved from 86.7 (SD = 5.8) at baseline to 0.2 (SD = 0.7) at 104 weeks (*F* = 750.5, *p* < 0.001).

## 4 Discussion

This modified Yoong technique represents a paradigm shift from traditional intra‐articular hydrodilation by targeting the SASD bursa at the RI. In this retrospective case series of 13 patients with frozen‐phase AC, the modified Yoong technique was associated with significant and sustained improvements over 2 years. Forward flexion improved from 76.6° to 178.8°, abduction from 60.6° to 178.8°, and external rotation from 31.3° to 78.0°. Mean VAS pain scores decreased from 5.6 to 0 by 52 weeks and remained at 0 through 104 weeks, while Quick DASH scores improved from 86.7 to 0.2. No complications were reported, and 92.3% of patients achieved clinical resolution by 12 weeks.

The rationale for this approach is anchored in the evolving understanding of AC pathoanatomy. While conventional wisdom has focused on glenohumeral joint capsule contracture, emerging evidence highlights that subacromial fibrosis and SASD bursal pathology contribute significantly to movement restriction. The SASD bursa is densely innervated with free nerve endings, making it a primary generator of pain in AC, which explains why mechanical distension of this space provides substantial symptomatic relief.

### 4.1 Outcome of Modified Yoong Technique

Our results demonstrate rapid and sustained improvements across all outcome measures. The mean VAS score decreased from 5.6 at baseline to 2.0 at 2 weeks, reaching zero by 52 weeks, with no recurrence of pain at 104 weeks. This pain trajectory compares favorably with published long‐term data: a prospective study of 202 patients undergoing ultrasound‐guided hydrodistension reported significant VAS reductions at 2 years, though diabetic patients in that cohort experienced worse outcomes and higher recurrence rates [8]. Similarly, another study reported better short‐term outcomes for hydro‐dilation compared to intra‐articular steroid injection [9]. The most common hydrodilation technique is done using the posterior approach [10]. A retrospective analysis showed immediate improvement in ROM following posterior approach hydrodilation of 20mls [11]. A systematic review also demonstrates that posterior shoulder hydrodilation provides short‐term and long‐term benefits in terms of pain and ROM [10]. However, the posterior approach requires longer needle lengths such as a spinal needle, especially for thick shoulders, and has a higher risk of poor needle visualization. Our finding that 53.8% of patients had underlying diabetes yet achieved complete pain resolution by 1 year suggests that the SASD‐targeted approach may overcome the poorer prognosis typically associated with diabetic AC.

The improvements in ROM were equally striking. Forward flexion increased from 76.6° to 178.8° by 52 weeks, and abduction improved from 60.6° to 178.8°, representing near‐complete restoration of functional movement. For context, a recent retrospective study of 150 patients receiving combined suprascapular nerve block and glenohumeral hydrodilatation reported median improvements of only 25 degrees in forward flexion and 19 degrees in external rotation at 3 months [12]. The good outcomes in our series may be attributable to the dual mechanism of the modified Yoong technique: (i) mechanical fenestration of the thickened CHL, which physically disrupts fibrotic contracture and (ii) high‐volume hydrodilation of the SASD bursa, which addresses extrinsic peritendinous adhesions that restrict gliding of the rotator cuff.

### 4.2 Advantages of Modified Yoong Technique

From a technical perspective, this modified approach offers several practical advantages over the original Yoong technique as in Table 2. First, needle placement superior to the CHL avoids the significant resistance encountered when penetrating a thickened, fibrotic ligament in the frozen phase, thereby reducing patient discomfort and procedural time. Second, the risk of inadvertent intra‐tendinous injection into the long head of the biceps or supraspinatus is minimized, as the needle tip remains within the SASD bursa, a forgiving plane with ample distensibility. Third, the procedure exhibits a relatively low learning curve, making it accessible to novice practitioners while maintaining a high success rate. No complications were reported in our series, consistent with the established safety profile of ultrasound‐guided shoulder injections.

**TABLE 2:** Showed the differences between Yoong technique and modified Yoong technique.

	Yoong technique	Modified Yoong technique
Approach	Glenohumeral injection	Subacromial–subdeltoid injection with fenestration of coracohumeral ligament
Target	Targeting anterior capsule	Targeting coracohumeral ligament and subacromial–subdeltoid fibrosis
Advantages	Hydrodilation of the capsule	Less painful
		Low learning curve
		Reduced injection time
Disadvantages	Painful as it requires to pass the thicken coracohumeral ligament	Does not target the anterior shoulder capsule
	Longer injection time	

The theoretical foundation for targeting the SASD bursa is supported by comparative studies on injection sites. A meta‐analysis by Shang et al. found no significant difference in pain relief or ROM outcomes when comparing intra‐articular to subacromial corticosteroid injections for AC [5]. This suggests that addressing the extrinsic peritendinous environment is at least as vital as treating the intrinsic joint space.

### 4.3 Strengths and Limitation

However, several limitations must be acknowledged. As a retrospective case series of only 13 patients, this study is inherently limited by selection bias, absence of a control group, and lack of standardized outcome assessment. The small sample size, predominantly female cohort (92.3%), and single‐center design severely limit generalizability. Moreover, the retrospective collection of secondary data introduces potential information bias, as outcomes were not originally recorded for research purposes. Manipulation under analgesic cover is known to improve ROM in AC by mechanically disrupting adhesions [13]. Therefore, the observed improvements in forward flexion, abduction, and external rotation cannot be attributed solely to the hydrodilation or corticosteroid injection. At minimum, the manipulation likely contributed additive or synergistic effects and is highly encouraged to be performed immediately after the procedure. Moreover, we acknowledge that there is potential for iatrogenic bleeding due to injury of the ascending humeral artery and risk of iatrogenic rupture of the long head of biceps tendon, although none of the complications occurred during 2 years follow up. Despite the preliminary nature of this study, several strengths warrant mention. First, this is the first report, to our knowledge, of 2‐year follow‐up outcomes for a modified Yoong technique targeting the SASD bursa rather than the glenohumeral joint, providing longitudinal data on durability of response. Furthermore, the absence of any complications in all 13 cases, despite inclusion of diabetic patients (53.8%) who are known to have higher procedural risks and poorer prognosis, supports the safety profile of this modified approach.

Despite these limitations, the modified Yoong technique presents a promising, technically less demanding alternative to existing hydrodilation methods. The dual therapeutic mechanism, CHL fenestration combined with SASD bursa hydrodilation, addresses both the intrinsic and extrinsic components of AC pathology. Future large‐scale, randomized controlled trials comparing this technique head‐to‐head with conventional intra‐articular hydrodilation are warranted.

## 5 Conclusion

This retrospective case series of 13 patients with AC in the frozen phase demonstrates that a modified ultrasound‐guided injection technique targeting the SASD bursa at the RI, combined with immediate postinjection manipulation, was associated with significant improvements in pain and ROM sustained over 2 years. No complications were observed.

Given the absence of a control group, the confounding effect of manipulation, and the small sample size, these findings should be considered preliminary. The modified technique offers a plausible alternative to conventional intra‐articular hydrodilation by avoiding needle traversal of a thickened CHL, which may theoretically reduce procedural difficulty and discomfort. However, direct comparative studies are required before claiming superiority. Larger, prospective randomized trials are needed to validate these observations and establish the technique's role in clinical practice.

## Author Contributions

**Nik Kamarul Arif:** conceptualization, writing – original draft, writing – review and editing, methodology, formal analysis. **Badrul Akmal Hisham Md Yusoff:** conceptualization, writing – original draft, methodology, writing – review and editing, formal analysis, supervision. **Muhammad Karbela Reza Ramlan:** conceptualization, writing – original draft, writing – review and editing, formal analysis. **Azwan Aziz Mohamad:** conceptualization, investigation, writing – original draft, writing – review and editing, methodology, formal analysis, supervision. **Norlelawati Mohamad:** conceptualization, investigation, writing – original draft, writing – review and editing, methodology.

## Funding

The authors have nothing to report.

## Consent

Written consent was taken prior to the study.

## Conflicts of Interest

The authors declare no conflicts of interest.

## Supporting information


**Video S1:** demonstrate the Modified Yoong technique for adhesive capsulitis. After confirmation of diagnosis, the coracohumeral ligament (CHL) is identified at the rotator interval, situated between the supraspinatus and subscapularis tendons. Following localization, 3–5 mL of local anesthetic is infiltrated into the subacromial–subdeltoid (SASD) bursa to provide analgesia. The CHL is then fenestrated using a 21‐gauge needle. Subsequently, the needle is withdrawn slightly back into the bursal space, and a 20 mL therapeutic mixture—comprising 4 mL of 1% lidocaine, 15 mL of 5% dextrose, and 1 mL of triamcinolone acetonide (40 mg)—is delivered. During injection, the clinician observes for the characteristic “pumping effect” to confirm proper fluid dispersion. Immediately following the injection, shoulder manipulation is performed, including forward flexion, abduction, and external rotation.

## Data Availability

The data that support the findings of this study are openly available in data Mendeley at https://data.mendeley.com/preview/x95ffwrjrv, reference 10.17632/x95ffwrjrv.1.
